# When Social Interactions Feel Less Meaningful: Age Differences in Solitude Preference in Daily Context

**DOI:** 10.1093/geroni/igaf122.4047

**Published:** 2025-12-31

**Authors:** Yoonseok Choi, Hansol Kim, Christiane Hoppmann

**Affiliations:** Simon Fraser University, Vancouver, British Columbia, Canada; Sogang University, Seoul, Republic of Korea; University of British Columbia, Vancouver, British Columbia, Canada

## Abstract

Solitude and social interactions have often been studied separately, each linked to distinct personality profiles (e.g., low vs. high extraversion). However, in daily life, these experiences may be interconnected as individuals transition between time alone and time with others. This study investigated whether lower-quality social interactions during the day are associated with a greater preference for solitude at the end of the day. Age moderation in this association was also examined. As younger individuals are more likely to engage in social interactions due to obligatory demands (e.g., work, childcare), we hypothesized that the proposed association is stronger among younger adults compared to older adults. Using daily life assessments up to 10 consecutive days, we assessed perceived meaningfulness of daily social interactions and preference for solitude at the end of the day in 128 individuals from South Korea and Canada (Age: M = 48.19 years, Range: 19-93; 67% female; 56% Asian, 32% White). Results based on multilevel modeling with ordinal outcomes showed that individuals who, on average, reported lower meaningfulness in social interactions exhibited a greater preference for solitude versus interacting or staying with others (between-person effect). The within-person association was moderated by age, such that younger individuals showed a stronger preference for solitude on days when their social interactions felt less meaningful. These findings underscore the role of social context and age in shaping motives for seeking solitude in daily life.

